# Continuous Production of Galacto-Oligosaccharides by an Enzyme Membrane Reactor Utilizing Free Enzymes

**DOI:** 10.3390/membranes10090203

**Published:** 2020-08-27

**Authors:** Teng Cao, Melinda Pázmándi, Ildikó Galambos, Zoltán Kovács

**Affiliations:** 1 Department of Food Engineering, Szent István University, 1118 Budapest, Hungary; Cao.Teng@hallgato.uni-szie.hu (T.C.); Pazmandi.Melinda@hallgato.uni-szie.hu (M.P.); 2 Department of Microbiology and Biotechnology, Szent István University, 1118 Budapest, Hungary; 3 Soós Ernő Water Technology Research and Development Center, University of Pannonia, 8200 Nagykanizsa, Hungary; galambos.ildiko@sooswrc.hu

**Keywords:** galacto-oligosaccharides (GOS), ultrafiltration (UF), enzyme membrane reactor (EMR), operational stability, lactose, prebiotics

## Abstract

Galacto-oligosaccharides (GOS) are prebiotic compounds widely used for their health-promoting effects. Conventionally, GOS is produced by the enzymatic conversion of lactose in stirred tank reactors (STR). The high operational costs associated with enzyme inactivation and removal might be reduced by the application of enzyme membrane reactors (EMR). In this study, we aimed to assess the potential of continuous GOS production by EMR using soluble Biolacta N5, a *Bacillus circulans*-derived commercial enzyme preparation. The steady-state performance of the EMR equipped with an ultrafiltration module was investigated as function of residence time (1.1–2.8 h) and enzyme load (17–190 U·g^−1^) under fixed operational settings of temperature (50 °C), pH (6.0), lactose feed concentration (300 g·kg^−1^), and recirculation flow-rate (0.18 m^3^·h^−1^). Results indicate that the yield of oligosaccharides with higher degree of polymerization (DP3-6) in STR (approx. 38% on total carbohydrate basis) exceeds that measured in EMR (ranging from 24% to 33%). However, a stable catalytic performance without a significant deterioration in product quality was observed when operating the EMR for an extended period of time (>120 h). Approx. 1.4 kg of DP3-6 was produced per one gram of crude enzyme preparation over the long-term campaigns, indicating that EMR efficiently recovers enzyme activity.

## 1. Introduction

Galacto-oligosaccharides (GOS) are non-digestible substances with prebiotic functions. The consumption of GOS results in the selective stimulus of probiotic bacteria (mostly *Lactobacilli* and *Bifidobacteria*) in the gut [1,2,3]. Increased ratio of probiotic strains in the gut microbiota positively influences human health by a multitude of mechanisms including inhibition of pathogens, stimulation of overall immune response, regulation of brain functions by metabolites and the possession of anti-cancer and anti-obesity effects [4,5,6,7]. Due to their health-promoting properties, the use of GOS in infant formulas, pharmaceutical, and nutraceutical products has been steadily increasing in the past decade. The global GOS market in 2018 was estimated at 600 million USD and is expected to increase to 960 million USD by the end of 2025 [8].

The industrial production of GOS is achieved via ß-galactosidase catalyzed enzymatic trans-galactolysis. The reaction results in a mixture of GOS fractions, non-reacted lactose, glucose, and small amounts of galactose as a by-product. The structure and degree of polymerization (DP) of GOS largely depends on the enzyme facilitating the synthesis. Industrial GOS production is carried out by GRAS- or QPS-certified enzymes that are usually of *Aspergillus oryzae*, *Kluyveromyces lactis*, or *Bacillus circulans* origin. The ß-galactosidase from *Bacillus circulans* has been favored by producers due to their thermostability and higher GOS yields compared to other commercial enzymes [9,10,11].

Traditionally, GOS are produced in batch-mode in stirred tank reactors (STR). This setup includes the inactivation and subsequent separation of the biocatalyst to achieve enzyme-free GOS products. These additional downstream processing steps increase the costs of GOS production. As the biocatalyst is the major cost factor of the STR process, prolonged application and potential re-use of ß-galactosidases is desired by GOS manufacturers [12,13,14].

Attempts to increase enzyme-life and simplify separation processes can be categorized into two approaches, the first strategy being enzyme immobilization and application of packed-bed and fixed catalytic membrane reactors. Although biocatalyst immobilization has been studied intensively in the last decade, the industrial application of this strategy is scarce. The main reason for this is the complexity and multimeric nature of industrial enzymes (such as ß-galactosidase) that make retaining enzyme activity during immobilization a challenging task [15,16].

The second alternative to batch processes is the application of ultrafiltration (UF)-assisted biocatalytic reactors, also known as enzymatic membrane reactors (EMR). In EMRs, continuous GOS synthesis is carried out by a free (or in some cases immobilized and suspended) enzyme in a stirred tank reactor coupled with an external membrane module. The membrane retains the enzyme in the reaction vessel, while saccharide fractions with lower molecular weights pass through it. As a result, continuous GOS synthesis and biocatalyst separation can be achieved simultaneously. The simple construction and straight-forward operation of EMRs makes this approach a promising alternative to traditional STR processes. However, some concerns arise with the application of EMRs, namely membrane fouling and loss of enzyme activity over long-term operations. Fouling can be addressed to a degree by proper process design and optimization while decrease in enzyme activity is counteracted by dosing fresh enzymes to the system. As the addition of enzymes increases cost, the application of stable enzymes and optimal operational parameters are important factors of sustainable EMR performance [13,15,17].

Previous attempts of GOS synthesis in EMRs have been carried out using commercial enzymes of *Kluyveromyces lactis* [18,19,20,21,22,23,24], *Aspergillus oryzae* [25,26,27], and *Bacillus circulans* [28]. Some attempts at GOS production have been made using enzyme-isolates of *Lactobacillus reuterii* [29], and thermostable ß-galactosidases isolated from archaea [30]. Reported GOS yields were mostly in the range of 20–30%, comparable to results obtained with STR processes. While these attempts demonstrate the applicability of EMRs in GOS synthesis, most of them investigate EMR performance in the short-term, as operational times are typically between 1–5 h, but no more than 24 h. Therefore, the two main factors of sustainable GOS synthesis in EMRs, long-term membrane fouling and enzyme inactivation, were not assessed.

To our knowledge, only two groups conducted long-term GOS experiments with 100 to 200 h of continuous EMR operation [24,30]. Both reported promising results: Ren et al. [24] reported no significant loss of activity after 96 h, while Petzelbauer et al. [30] reported half-lives of the utilized enzymes to be about 7 days. Moreover, Cordova et al. [26] calculated the half-life of a commercial *Kluyveromyces lactis* enzyme to be 231 h, although the process was only operated for 22 h [26]. These observations on enzyme stability suggest great potential of the free-enzyme EMR processes.

So far, no attempts have been made to investigate the potential of commercially available *Bacillus circulans* enzymes for GOS production in long-term EMR setups. As mentioned earlier, these enzymes have received great attention due to favorable operational properties (relative thermotolerance and high GOS yields compared to other commercial enzymes). Our current knowledge about the behavior of *Bacillus circulans* ß-galactosidase in EMRs is limited. Warmerdam [31] investigated the stability of Biolacta N5, a commercially available *B. circulans*-derived ß-galactosidase, using an ONPG activity assay in batch setup. An increased stability of the enzyme was observed at high lactose concentrations, and the half-life of Biolacta N5 using an initial lactose concentration of 30 *w*/*w*% was reported to be 29, 29, and 16 h, for 25, 40, and 60 °C, respectively [31].

As lactose concentration is maintained at a high level during EMR operation, this technique might be favorable in terms of enzyme activity, stability, and related GOS production. The aim of this study was to investigate the performance of the GOS synthesis catalyzed by a commercially available *B. circulans* ß-galactosidase (Biolacta N5) in an EMR setup. Short- and long-term experiments were carried out to assess performance in terms of GOS yield and operational stability.

## 2. Materials and Methods

### 2.1. Materials

Biolacta N5 (Amano Enzyme Inc., Nagoya, Japan), a β-galactosidase derived from *Bacillus circulans*, was used to convert lactose into GOS. The activity of the crude enzyme preparation used in this study was 19,062 ± 2457 U·g^−1^, as determined by the activity assay detailed in Section 2.2. Lactochem Fine Powder, a pharmaceutical-grade α-lactose monohydrate manufactured by FrieslandCampina Domo B. V. (Amersfoort, The Netherlands), was used as substrate in all experiments with the exception of the long-term investigations. For the long-term campaigns (see Section 2.8), Lactopure Regular Power 150 M (FrieslandCampina Domo B. V., Amersfoort, The Netherlands) was used, which is a whey-derived, food-grade lactose preparation with a typical lactose content of 99.7%.

### 2.2. Determination of Enzyme Activity

The β-glycosidase activity of Biolacta N5 was measured by using 300 g·kg^−1^ lactose as a substrate and an enzyme concentration of 0.3 g·kg^−1^ at 50 °C and pH 6.0 adjusted by NaOH. The reaction was stopped after 40 min incubation time by a 30 min heat treatment at 90 °C. The disaccharide fraction (DP2) in the heat-treated sample was measured by HPLC, as described in Section 2.9. One unit of enzyme activity (U) was defined as the amount of crude enzyme that converts 1 μmol of DP2 per minute under the above assay conditions.

### 2.3. Batch Conversion

The enzymatic conversion of lactose by Biolacta N5 was performed in batch fashion by using a C-MAG HS7 hotplate magnetic stirrer by IKA-Werke GmbH & Co. KG (Staufen, Germany) equipped with an electronic contact thermometer for precise temperature control. The reaction liquor was prepared using a dosage of 5.7 U·g^−1^ of crude enzyme preparation and an initial lactose concentration of 300 g·kg^−1^. The reaction was carried out in triplicate at 50 °C and at pH 6.0 in 200 g units by gently stirring the reaction liquor at 60 rpm. Samples were taken periodically during the reaction and kept at 90 °C for 30 min prior to HPLC analysis to deactivate enzymes.

### 2.4. Enzyme Membrane Reactor (EMR)

**Figure 1:**
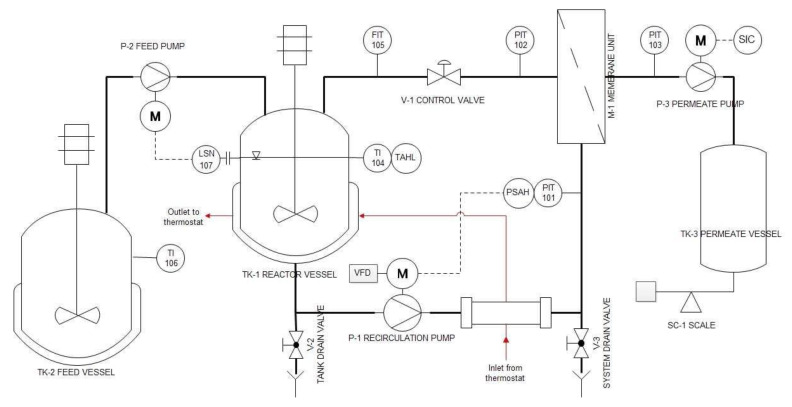
Piping and instrumentation diagram of continuous ultrafiltration-assisted enzymatic reactor (EMR).

The flowsheet of the semi-pilot scale EMR used for the continuous production of GOS is shown in Figure 1. The EMR consists of a stirred-tank reactor (TK-1) and an external ultrafiltration module (M-1). The set-up allows for the control of the recirculation flowrate, the retentate pressure, the temperature, the permeate flow, and the liquid level in TK-1 during operation.

The enzymatic catalysis took place in the 4 L reaction vessel (TK-1) using soluble enzymes. A Hydra-Cell D-10 diaphragm pump (Wanner Engineering, Inc., Minneapolis, MN, USA) was employed as recirculation pump (P-1). P-1 circulated the reaction liquor through the UF module. The recirculation flowrate was set by the frequency drive (VFD) and measured with the magnetic-inductive flow meter SM6000 (FIT) manufactured by IFM electronic Gmbh (Essen, Germany). The retentate-side pressure was adjusted manually to the desired value by the control valve V-1, and measured by the pressure gauge PIT-102 (SUKU 6850, SUKU GmbH, Lichtenau OT Garnsdorf, Germany). The permeate was collected in the permeate vessel TK-3, and its weight was monitored by the scale SC-1 (FKB 30K1A, KERN&SOHN GmbH, Balingen, Germany). The desired value of the permeate flow was set by adjusting the speed of the peristaltic pump P-3 (323 SD, Watson Marlow Inc., Wilmington, MA, USA). The permeate-side pressure was monitored by the pressure gauge PIT-103 (SUKU 6850, SUKU GmbH, Lichtenau OT Garnsdorf, Germany). The permeate flux was determined manually by measuring the weight of the collected permeate samples and the respective time. The substrate solution consisting of 300 g·kg^−1^ lactose was stored in the 50 L jacketed stainless-steel feed vessel (TK-2). Fresh substrate solution was supplied from TK-2 into TK-1 by the feed pump (P-2) in a quasi-continuous manner. The peristaltic pump P-2 (OEM M1500, Verder Hungary Kft, Budapest, Hungary) was regulated by a Nivocont KKH-212-5 compact conductive level switch installed in TK-1 to ensure a constant volume in the reactor TK-1. The substrate solution in TK-2 was thermostated at 50 °C with a built-in electrical heating unit. The temperature of the reaction liquid was kept at 50 °C by circulating warm water generated by a Julabo VC (Julabo GmbH, Seelbach, Germany) laboratory thermostat through a double pipe heat exchanger and the jacket of TK-1. The actual temperature in the reactor was measured by the digital temperature sensor TI-101 purchased from Dostmann GmbH, Wertheim-Reich olzheim, Germany. In the stainless-steel housing of M-1, a 2′′ spiral-wound element was installed. A ST-2B-1812PHT-F element in sanitary full-fit design was purchased from Synder Filtration Inc, Vacaville, CA, USA. The 10 kDa UF membrane was made of a polyethersulfone active layer cast on polypropylene backing material and had a filtration area of approx. 0.37 m^2^.

### 2.5. Terminology

The following measures were used in this study:

- Relative mass fraction ($w_{i}$) was calculated as the ratio of the mass of a saccharide fraction *i* ($m_{i}$) to the total mass of saccharides present in the solution:
  (1) $$w_{i}=\frac{m_{i}}{\sum m_{i}}$$
- Relative mass percentage was the relative mass fraction ($w_{i}$ ) expressed in percentage;
- Residence time (*τ*) was given as the weight of the reaction liquor in the reactor (*m_R_*) divided by the mass flow rate of the permeate (*q*):
  (2) $$\tau =\frac{m_{R}}{q}$$
- Yield (*Y*) was defined as the concentration of the generated DP3-6 fractions ($c_{DP3-6})$ divided by the concentration of lactose in the feed ($c_{L}$ ):
  (3) $$Y=\frac{c_{DP3-6}}{c_{L}}\;\times 100\%$$
- Biocatalyst productivity (*P*) was the total quantity of DP3-6 formed by one unit of crude enzyme preparation per hour:
  (4) $$P=\frac{c_{DP3-6}}{c_{E}\cdot \tau }$$

### 2.6. Preliminary Filtration Tests

Two preliminary filtration tests were carried out, as described below in Section 2.6.1 and Section 2.6.2. Both tests were performed in total-recycle mode, i.e., recirculating both retentate and permeate streams into the reactor. The recirculation flowrate of the retentate was set to 0.18 m^3^·h^−1^. The experiments were performed with 2 kg of process liquid consisting of 30 *w*/*w*% lactose. The pH was set to 6.0 using NaOH, and the temperature was kept at 50 °C. The permeate flux was monitored during the test runs. No samples for chemical analysis were taken. 

#### 2.6.1. Pressure-Scan

Ten g·kg^−1^ Biolacta N5 was dosed into the process liquid. The transmembrane pressure was raised stepwise from 1 to 4 bar. Permeate flux was monitored.

#### 2.6.2. Determination of Limiting Flux

One g·kg^−1^ Biolacta N5 was dosed into the process liquid. The transmembrane pressure was set to 4.0 bar. The permeate flux was monitored until it reached a steady value (ca. after 1 h). Then, the enzyme load was increased, and the same procedure was repeated. The (quasi) steady value of permeate flux was registered for enzyme loads in the range between 1 and 120 g·kg^−1^.

#### 2.6.3. Membrane Cleaning

Before each test with the process liquor, the permeability of deionized water was measured. After the tests with the process liquor, a four-step procedure was performed as follows:

1. The membrane plant was drained and flashed several times with deionized water.
2. Membrane cleaning was carried out by circulating a NaOH solution (pH = 10–11) for 1–2 h at 40–50 °C under 0.5–1 bar pressure.
3. The plant was drained and flushed several times with water to remove the cleaning agent.
4. Permeability of the cleaned membrane was measured with DI water. In certain cases, when the original permeability of the membrane (<25%) was not recovered by the alkaline cleaning procedure, then additional cleaning with citric acid and/or Ultrasil (Ecolab, Paul, MN, USA) was performed (1 *w*/*w*%, 40–50 °C, 0.5–1 bar, 0.5–1 h).

### 2.7. Short-Term Catalytic Runs

Eight short-term tests were carried out by operating the EMR in continuous fashion. The experiments were performed by varying the enzyme load (between 5.7 and 190.6 U·g^−1^) and the permeate flow (between 0.8 and 1.8 kg·h^−1^) under otherwise identical conditions. For all tests, a 30 *w*/*w*% lactose solution was prepared, and its pH was set to 6.0 using NaOH. Two kg of the lactose solution was introduced into the reactor, and the remaining solution was stored in the thermostated substrate tank. The reactor was thermostated at 50 °C, and certain amount of Biolacta N5 was dosed into the vessel. After enzyme dosage, the circulation pump was put into operation using a cross flowrate of 0.17 ± 0.01 m^3^·h^−1^. The retentate pressure was set to 0.5 bar with the pressure adjusting valve. Then, the rotation of the permeate pump was adjusted to generate constant permeate flowrate, resulting in a constant residence time. The EMR was operated in a continuous manner, typically for 6 to 9 h. During the whole process, fresh substrate solution was continuously fed into the reactor at the same rate as the enzyme-free product was removed to ensure a constant volume in the reactor. Temperature, retentate- and permeate-side pressures, permeate flowrate, and the total mass of collected permeate were monitored during the process runs. Samples were periodically taken from the permeate stream (i.e., from the enzyme-free product stream) for carbohydrate analysis. Membrane cleaning was performed as previously described in Section 2.6.3.

### 2.8. Long-Term Catalyst Runs

Two long-term experiments, denoted as L1 and L2, were performed under identical operational conditions for an extended period of time (over 100 h). In both runs, the enzyme concentration in the reactor was set to 95.3 U·g^−1^. The retentate pressure was adjusted to 1.0 bar. The permeate pump was set to generate a constant flowrate of 1.1 kg·h^−1^, resulting in a residence time of 1.8 h. Other operation parameters were the same as in the short-term runs (2 kg reaction liquid, 30 *w*/*w*% feed lactose concentration, 50 °C, pH 6.0, 0.17 m^3^·h^−1^ recirculation flowrate rate). Samples were taken periodically from the permeate stream for HPLC analysis. At each sampling, 3 samples were taken. Samples taken from L1 were analyzed by HPLC without pre-treatment. In L2, one sample was subject to heat deactivation (90 °C, 30 min), one to acid deactivation (HCl), and one was analyzed without deactivation pre-treatment. Membrane cleaning was performed as previously described in Section 2.6.3.

### 2.9. Statistical Test

The relationship between the steady-state carbohydrate composition and the operational factor (*τ* × *c_E_*) was analyzed by nonlinear regression. In order to derive a quantitative relationship, an arbitrarily chosen empirical model was fitted to the observed values of the reaction products (DP3-6, glucose, and galactose) as a function of operational parameters such that:

(5) $$w_{i}=\frac{b_{1}\tau c_{E}}{b_{2}\tau c_{E}+1}+\;\varepsilon$$

where $w_{i}$ is the relative mass percentage of the individual saccharide fraction at steady-state, *b*_1_ and *b*_2_ are model coefficients, *τ* is the residence time, *c_E_* is the enzyme concentration, and ε is the error term. In the case of the DP2 fraction, the regression model was used in the following form:

(6) $$w_{DP2}=100-\frac{b_{1}\tau c_{E}}{b_{2}\tau c_{E}+1}+\;\varepsilon$$

The statistical assessment was performed with the statistics and curve fitting toolboxes implemented in Matlab R2015a (The Mathwork Inc., Natick, MA, USA).

### 2.10. High Performance Liquid Chromatography

Carbohydrate composition (glucose, galactose, and DP2-6 fractions) of samples was determined by the HPLC method, as previously described in [32]. In brief, a Thermo Separations HPLC system equipped with a Shodex R-101 refractive index detector (Showa Denko Europe GmbH, Munich, Germany) and a N2000 Chromatography Data System from Science Technology (Hangzhou) Inc. (Hangzhou, China) was used, employing an RNM carbohydrate 8% Na^+^ 300 × 7.8 (Phenomenex, Torrance, CA, USA) analytical column at 50 °C at 0.2 mL·min^−1^ with a prefiltered (2 µm) DI water as mobile phase. Specification of standards used for peak identification can be found in [32]. Samples taken from the product (= permeate) stream of the EMR were not subject to heat deactivation prior to HPLC analysis unless otherwise stated. Samples collected during batch experiments were subject to heat treatment (90 °C, 30 min) prior to HPLC analysis. The HPLC analysis was based on the degree of polymerization, a further structural characterization of the generated DP2–5 fractions was out of the scope of this study.

## 3. Results and Discussion

### 3.1. Preliminary Filtration Experiments

The aim of the preliminary filtration tests was to investigate the filtration performance of the UF membrane when challenged with the process liquor. More specifically, the effect of the trans-membrane pressure, enzyme load, and operational time on the permeate flux was determined. Over the long-term operation of EMR, membrane fouling and enzyme activity losses were expected. The latter could be compensated by dosing fresh enzymes gradually into the reactor to ensure a constant product quality. Both fouling and increasing enzyme load resulted in a flux decline over time. Our aim was to identify the set of design parameters of the EMR (such as membrane area, reactor volume, residence time, and trans-membrane pressure), at which a stable level of the desired product flow over a long period of operation was ensured.

#### 3.1.1. Pressure-Scan

**Figure 2:**
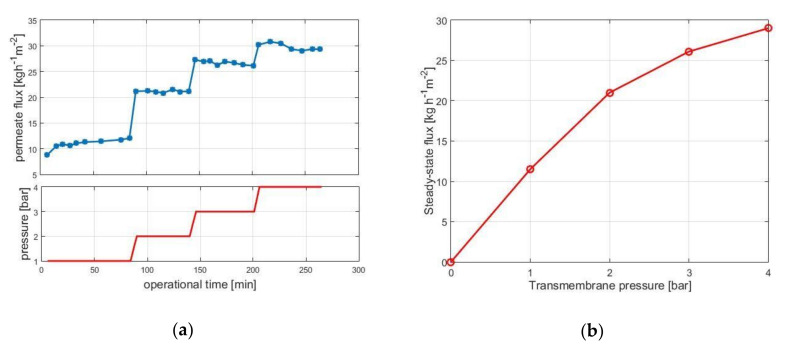
Permeate flux as function of operational time for various transmembrane pressures (**a**) and quasi steady-state flux values versus pressure (**b**). Operational conditions: 30 *w*/*w*% lactose concentration, 10 g·kg^−1^ enzyme load, pH 6.0, 50 °C, 0.18 m^3^·h^−1^ crossflow rate, 10 kDa UF membrane.

Figure 2a shows the permeate flux as a function of operation time for transmembrane pressures of 1, 2, 3, and 4 bar. The decay in permeate flux over operation time is small, indicating that fouling is not pronounced. The (quasi) steady state flux values are then plotted against the transmembrane pressure in Figure 2b. Results indicate that under approx. 4 bar the system operates in the so-called pressure-dependent regime, i.e., flux can be increased by increasing the transmembrane pressure.

Over approx. 4 bar, little increase in flux can be achieved by increasing the pressure; this is the so-called pressure-independent regime. The pressure-independent regime is known for pronounced fouling, and the pressure-dependent regime is usually recommended for UF operations.

#### 3.1.2. Limiting Flux

The limiting flux model provides a simple and convenient procedure for interpreting experimental UF data [33]. During filtration, the rejected solutes tend to accumulate on the membrane surface and form a concentration polarization layer. At steady state, the quantity of solutes conveyed by the solvent to the membrane is equal to those that diffuse back. In most UF applications (e.g., in [34,35]), the permeate flux becomes essentially independent of pressure at high pressures. A physical model describing this phenomenon is known as the limiting flux (or traditionally, *gel polarization*) model. This model assumes that the protein concentration at the membrane surface reaches a limiting concentration under conditions where high protein retention and membrane fluxes are present. At fixed hydrodynamic conditions, the flux can be related to the limiting concentration as:

(7) $$J_{lim}=kln(\frac{C_{lim}}{C_{b}})$$

where *k* is the mass transfer coefficient under polarized-layer-controlled conditions, *c_b_* is the concentration of enzyme in the bulk, and *c*_lim_ is the limiting concentration. This latter term is historically referred to as gel concentration; however, the limiting flux generally occurs independently of any supposed gelation effects. It has been shown that *c*_lim_ is rather a phenomenological variable than a true physical property the solution [36]. 

**Figure 3:**
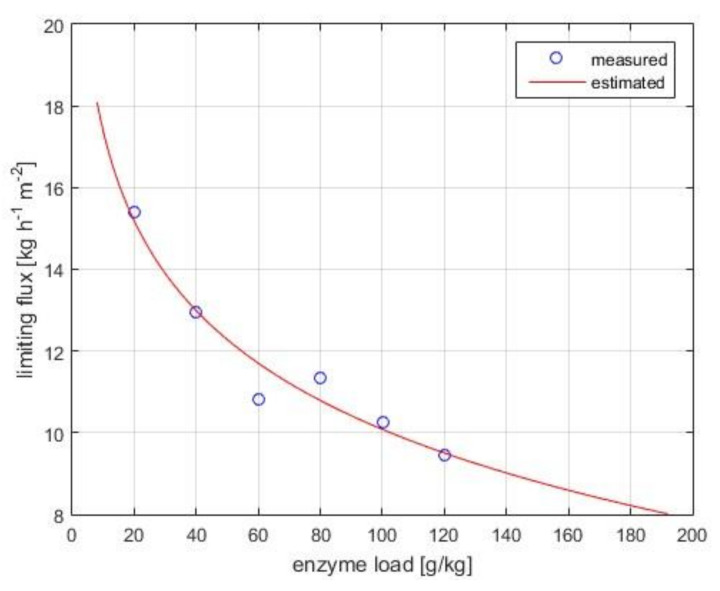
Limiting flux versus bulk enzyme concentration in the pressure-independent zone. Operational conditions: 4 bar, 30 *w*/*w*% lactose concentration, pH 6.0, 50 °C, 0.18 m^3^·h^−1^ crossflow rate, 10 kDa UF membrane. Measured and estimated (using Equation (1)) data are illustrated with symbols and continuous lines, respectively.

Figure 3 shows the experimental flux data collected in the pressure-independent regime. The limiting flux model (Equation (7)) fits well the observed data (*R*^2^ = 0.985). The mass transfer coefficient under polarized-layer controlled conditions, k, and the limiting concentration, *c*_lim_, are computed as 3.17 kg·h^−1^ m^−2^ and 2405 g·kg^−1^, respectively.

### 3.2. Catalytic Performance

#### 3.2.1. Batch Conversion in STR

**Figure 4:**
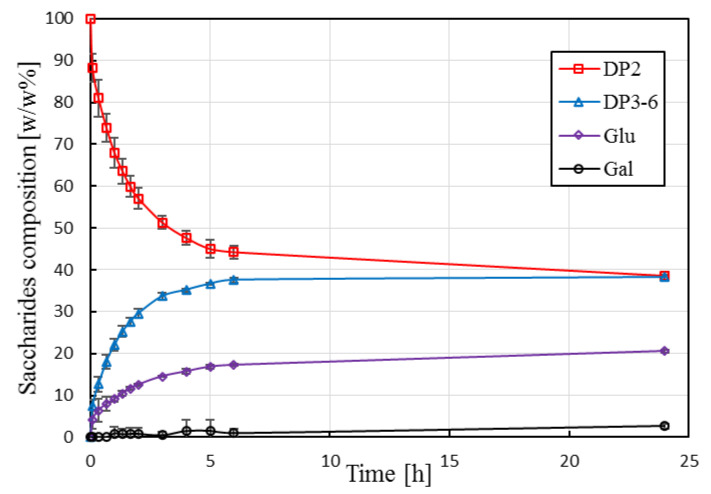
Saccharide composition as function of reaction time in stirred-tank reactor. Operational conditions: 30 g·kg^−1^ initial lactose concentration, 19.1 U·g^−1^, pH 6.0, 50 °C, 60 rpm. Mean values and standard deviation of triplicate measurements are shown. Continuous lines are guides for the eye.

The carbohydrate profiles for batch enzymatic conversion are shown in Figure 4.

Lactose is converted into GOS, glucose, and a small amount of galactose. The relative mass percentage of DP3-6 fractions reaches a plateau at ca. 38 *w*/*w*% after about 6 h. The presented results are in good agreement with literature data reported for Biolacta N5 applying similar reaction conditions [31,37]. Previous studies have also shown that a further increase in initial lactose concentration (above ca. 30 *w*/*w*%) does not lead to an increased yield [32,37]. The space–time yield, which is the amount of product (DP3-6) obtained per hour for one kg of reactor content, was calculated as 18.8 g·kg^−1^·h^−1^ (based on 6 h processing time and normalized to 1 kg reactor content). It should be noted that batch production scheme, unlike continuous production in EMR, requires additional processing steps including heat treatment for enzyme inactivation and subsequent downstream operations to remove inactivated enzymes. These time-consuming processing steps increase operational costs of STR-based processes.

#### 3.2.2. Short-Term Runs in EMR

**Figure 5:**
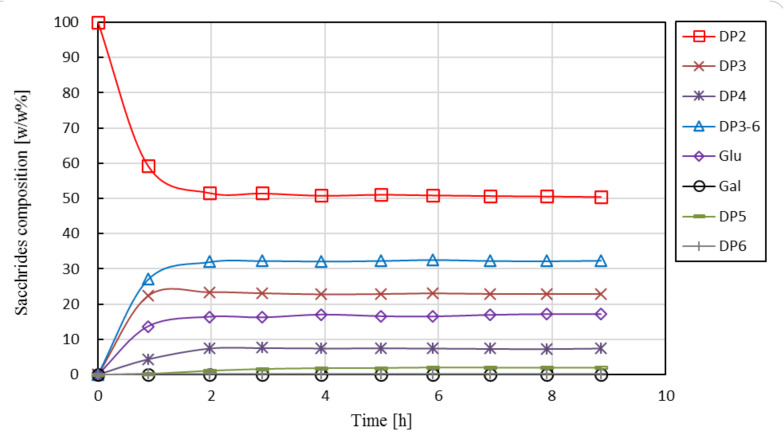
Saccharide composition in permeate of the enzyme membrane reactor as function of operational time during Run No2. Operational conditions: 30 *w*/*w*% feed lactose concentration, 30 *w*/*w*% initial lactose concentration, 19.1 U·g^−1^ enzyme load, pH 6.0, 50 °C, 2.2 h residence time, 0.18 m^3^·h^−1^ cross-flow rate, 0.5 bar retentate-side pressure, ca. 0.40–0.45 bar permeate-side pressure, 10 kDa UF membrane.

Eight short-term experiments were carried out to investigate the steady-state performance of the EMR as function of enzyme load and residence time. As an illustrative example, the change in the saccharides’ composition in the product stream over processing time is shown in Figure 5 for a typical process run (No. 2).

Short-term tests were carried out using 2 kg of reaction liquor and setting the permeate flow to a constant value by adjusting the speed of the permeate pump. The permeate flow was varied in the range of 0.9 to 1.9 kg·h^−1^ in order to adjust the desired residence time for each experiment. The carbohydrate profiles were monitored during the test runs. The steady-state saccharides’ composition of the short-term runs is summarized in Table 1.

The degree of conversion is a function of both the enzyme dosage (*c_E_*) and the residence time (*τ*). At *c_E_* = 0 or *τ* = 0 settings, no substrate conversion takes place. As expected, an increase in the enzyme dosage resulted in a higher conversion of lactose. In addition, an increase in residence time enhanced the lactose conversion. In order to express and to visualize the combined effects of both parameters, we use the product of enzyme load and residence time (*τ* × *c_E_*, in U·h·kg^−1^) as a simple measure of the applied settings that determines product quality. With an increase in *τ* × *c_E_* from 21 to 540 U·h·g^−1^, a gradual decline in productivity from approx. 3.4 × 10^−3^ to 0.2 × 10^−3^ g of DP3-6 per hour and unit enzyme activity was observed under the studied operational settings.

**Figure 6:**
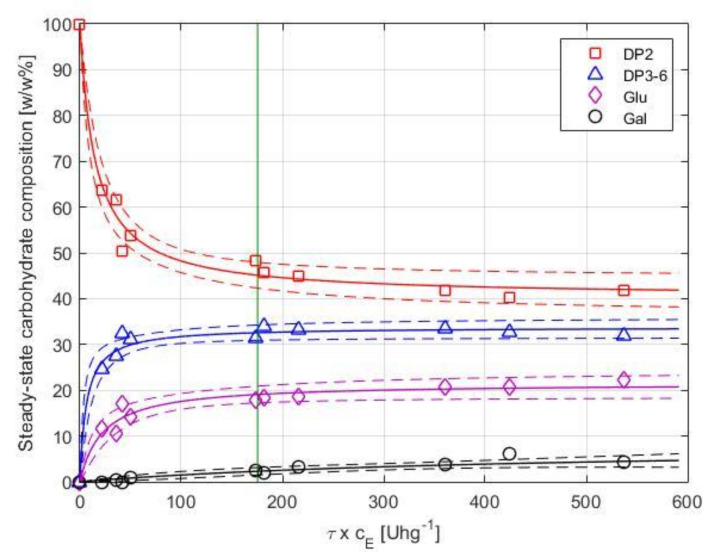
Steady-state saccharides’ composition as function of *τ* × *c_E_* for short-term runs. Experimental data, fitted regression models, and simultaneous 95% confidence bounds are illustrated with symbols, solid lines, and dashed lines, respectively. Data obtained for long-term runs L1 and L2 at 8 h of operation is highlighted with a vertical line at *τ* × *c_E_* = 175.4 U·g^−1^.

The product composition as a function of *τ* × *c_E_* is shown in Figure 6. Equations (5) and (6) were fitted to the observed concentrations of the individual carbohydrate fractions, separately. Nonlinear regression was performed using the residence time (*τ*) and enzyme load (*c_E_*) as predictors and the relative mass percentages of the saccharide fractions ($w_{i}$) as response variables. Results of the analysis are summarized in Table 2. The model estimates and simultaneous 95% confidence bounds are shown in Figure 6.

As shown in Figure 6, the DP3-6 fraction initially increases by increasing *τ* × *c_E_* and flattens at *Y* ≅ 33 *w*/*w*%. Above ca. 200 U·h·g^−1^, no significant enhancement in yield can be achieved, and a further increase of *τ* × *c_E_* results in the formation of hydrolysis by-products. The DP3-6 yield of the EMR (25–33%) is less than that of the STR (38%).

#### 3.2.3. Long-Term Performance

Two long-term runs (Run L1 and L2) were carried out under identical operational conditions. In both runs, the EMR was operated continuously for over 120 h. A total of ca. 40 kg of lactose (i.e., roughly 130 kg of fresh lactose solution) was utilized as feed in each run, and accordingly, ca. 130 kg liquid product was withdrawn from the EMR as the permeate. Given that the enzyme dosage in the EMR was approx. 5 g/kg, a DP 3-6 production of approx. 1.4 kg per 1 g of crude enzyme preparation was reported for the whole campaign.

**Figure 7:**
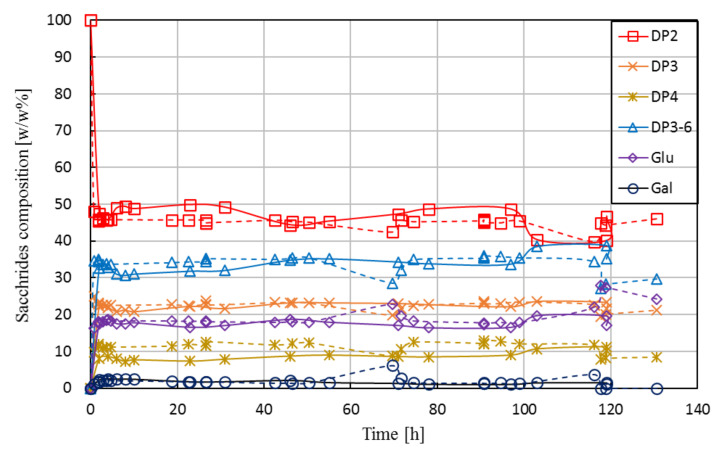
Saccharides’ composition in permeate as function of operational time in enzymatic membrane reactor for both Run L1 (solid line) and Run L2 (dashed line). Operational conditions: 30 *w*/*w*% feed lactose concentration, 30 *w*/*w*% initial lactose concentration, 95.3 U·g^−1^ enzyme load, pH 6.0, 50 °C, ca. 1.8 h residence time, 0.16 m^3^·h^−1^ cross-flow rate, 1.0 bar retentate-side pressure, 0.7–0.2 bar permeate-side pressure, 10 kDa UF membrane.

Figure 7 shows the saccharides’ composition in the product stream as a function of operational time for Run L1 and Run L2.

The campaigns delivered comparable results; data obtained from L2 were in good agreement with that of L1. In addition, the steady-state composition achieved during the long-term runs (mean value calculated from samples taken at around 5–10 h) fit well with the tendency observed for short-term runs, as shown earlier in Figure 6 (see vertical line). The experiments were performed by maintaining both the reactor volume and the permeate flow-rate (and thus, the residence time) at a constant level. A constant volume in the reactor was ensured by level control using an on/off triggered feed pump by a level sensor, as described in Section 2.4. A constant value of permeate flow was achieved by adjusting the retentate pressure to 1 bar by the retentate-side valve and the permeate flow to ca. 1.1 kg·h^−1^ by the permeate pump. During the long-term tests, the permeate pressure gradually decreased from ca. 0.7 to 0.2 bar, i.e., the transmembrane (net) pressure increased from 0.3 to 0.8 bar, indicating a slow development of fouling. Note that Lactopure contains small amounts of whey residues (e.g., proteins) that may be rejected partly or completely by the UF membrane. Although the accumulation of such compounds in the system likely contributed to fouling development, it did not cause the flux to drop below the set value. In case of both short-term and long-term investigations, the membrane was successfully regenerated after the experiments. In most instances, water permeability (≈18 ± 3.6 kg·h^−1^·m^−2^) was restored by applying alkaline cleaning. In some cases, original flux was only recovered by applying additional cleaning steps, following the procedure described in Section 2.6.3. 

When comparing the results of long-term runs with those of the STR experiment, we see that STR outperforms EMR in terms of yield (38% vs. 33%) and biocatalyst productivity (3.3 × 10^−3^ vs. 0.6 × 10^−3^ g·U^−1^·h^−1^). However, the amount of product (DP3-6) obtained per hour for one kg of reactor content was calculated as 52.7 g·kg^−1^·h^−1^ for the long-term runs which compares favorably to STR (18.8 g·kg^−1^·h^−1^). In addition, the total amount of DP3-6 produced by one gram of enzyme preparation was considerably higher in EMR (ca. 1.4 kg g^−1^) than in STR (ca. 0.4 kg g^−1^) under the studied operational settings.

One of the main reasons for repeating the experiment of Run L1 was to prove that the obtained data on conversion was correct. In order to exclude the possibility of any remaining enzyme activities in the samples taken from the permeate (caused by, e.g., undesired enzyme leakage), samples from L2 were subject to pre-treatment (none/heat/acidic) before HPLC analysis. The pre-treatment, however, had no effect on the HPLC results.

A stable degree of conversion measured for such an extended period of time, as reported in Figure 7, is surprising. As previously noted, no reports are available on the operational stability of Biolacta N5 in EMR. To the best of our knowledge, the only available data on half-life (29, 29, and 16 h, for 25, 40, and 60 °C, respectively) was measured by ONPG assay in batch set-up [31]. Based on that, we expected a pronounced decline in conversion over time. Recall that the *τ* × *c_E_* factor in the long-term runs was adjusted to ca. 176 U·h·g^−1^. Obviously, this setting does not necessarily represent optimal operational conditions and results in best-performing operation. However, we hoped this setting would give us a closer insight into the activity losses occurring during a long-term run. According to [31], we expected a half-life of about 24 h. Starting the long-term run with an enzyme dosage of 95 U·g^−1^ (*τ* × *c_E_* = 176 U·h·g^−1^), this means that the concentration of active enzymes was expected to drop to 48 U·g^−1^ (*τ* × *c_E_* = 88) after approximately one day of operation. By the end of the experiment, at ca. 120 h, *τ* × *c_E_* was expected to reach the value of 6. As illustrated in Figure 6, such a decline in activity would result in a considerable decrease in the degree of conversion that could be detected by HPLC by ease. Figure 6 reports the proven output of EMR for any given value of *τ* × *c_E_*. Thus, by following the expected decrease in *τ* × *c_E_* from our starting situation (*τ* × *c_E_* = 176) to *τ* × *c_E_* = 6, one can read the corresponding degree of conversion from the vertical axis in Figure 6. Presuming a half-life of 24 h, the regression models (Equations (5) and (6)) estimated a dramatic drop in product quality after 5 days of operation, resulting in a saccharide composition of 84.1 ± 4.4 *w*/*w*% DP2, 15.5 ± 5.2 *w*/*w*% DP3-6, 4.2 ± 2.0 *w*/*w*% glucose, and 0.1 ± 0.1 *w*/*w*% galactose. Due to the stable EMR performance, HPLC data from L1 and L2 cannot be used for extrapolation to determine true value of enzyme half-life. Exact value for half-life could likely be determined by performing longer experiments at scaled down EMR size to minimize raw material costs or by repeated experiments at suboptimal operational settings (i.e., performing tests at lower initial value of *τ* × *c_E_*).

## 4. Conclusions

An ultrafiltration-assisted membrane reactor utilizing free enzymes was designed and equipped with the necessary control system to operate at a constant product flow. Preliminary filtration tests with the reaction liquor were performed to characterize the flux performance of the UF membrane related to crucial scale-up parameters. The dependence of transmembrane pressure and enzyme load on the permeate flux was experimentally determined. During our experimental investigations, no irreversible losses in water permeability caused by fouling were observed. The membrane was successfully regenerated by the reported cleaning procedure.

A series of short-term experiments were conducted by operating the EMR in continuous fashion, typically for 6–9 h. These tests were performed to determine the steady-state performance of the EMR in terms of yield and productivity. The effect of residence time (in the range from 1.1 to 2.8 h) and enzyme load (17–190 U·g^−1^) on the catalytic performance was investigated under fixed operational settings of temperature (50 °C), pH (6.0), lactose feed concentration (300 g·kg^−1^), and recirculation flow-rate (0.18 m^3^·h^−1^). Results indicated that the DP3-6 yield in EMR was slightly below the yield measured in STR (approx. 38% on total carbohydrate basis). In EMR, the yield increased from 24% to 33% and the productivity decreased from approx. 3.4 × 10^−3^ to 0.2 × 10^−3^ g·h^−1^·U^−1^ by increasing the operational factor, *τ* × *c_E_*, from 21 to 540 U·h·g^−1^.

Generally, the EMR is known as a convenient way of a continuous production process with enzyme reuse. However, enzyme stability is a crucial factor for EMR. The stability of the enzyme affects the amounts of fresh enzymes to be dosed into the reactor in order to compensate activity losses over time, and thus, to ensure constant product quality. An experiment run in duplicate was carried out to investigate the catalytic performance of the EMR for an extended period of time. In both test runs, the EMR showed a stable operational performance in terms of yield and conversion without a significant deterioration in product quality for over 120 h. As for methodological limitations, the exact amount of activity losses was not determined. However, analysis revealed that operational stability of the enzyme in EMR is considerably higher than that previously reported for Biolacta N5 in STR process [31]. During the campaigns, an average of approx. 1.4 kg of DP3-6 was produced by one gram of crude enzyme preparation. These results suggest that EMR might serve as a promising alternative to conventional batch production scheme, especially considering the high price of the biocatalysts.

## Figures and Tables

**Table 1:** Steady-state saccharides’ composition in *w*/*w*% for short-term EMR runs. The composition obtained for batch process (at 6 h) is indicated for comparison purpose.

Component	No3	No5	No2	No7	No4	No6	No1	No8	Batch
*τ* [h]	1.1	2.1	2.2	2.6	1.1	2.1	2.2	2.8	6.0
*c_E_* [U·g^−1^]	19.1	17.3	19.1	19.1	190.6	173.4	190.6	190.6	5.7
*τ* × *c_E_* [U·h·g^−1^]	21.5	36.1	41.9	49.8	215.4	360.8	423.6	537.5	34.3
*P* [g·h^−1^·U^−1^] × 10^−3^	3.42	2.28	2.32	1.87	0.46	0.28	0.23	0.18	3.28
DP2	63.8	61.7	50.5	53.8	45.0	41.9	40.2	41.7	44.2
Glu	11.7	10.5	17.1	14.3	18.6	20.8	20.8	22.2	17.2
Gal	0.0	0.4	0.0	0.9	3.3	3.8	6.2	4.2	1.0
DP3	19.7	22.0	22.9	22.9	21.6	20.6	20.6	20.7	25.0
DP4	4.4	5.4	7.5	6.8	7.9	8.8	8.3	8.6	10.5
DP5	0.6	0.0	1.9	1.3	2.7	4.0	3.7	2.6	2.0
DP6	0.0	0.0	0.2	0.0	1.0	0.0	0.1	0.0	0.1
DP3-6	24.6	27.4	32.4	31.0	33.2	33.4	32.8	31.9	37.6

**Table 2:** Estimated regression coefficients and goodness of fit statistics.

Response Variable	Model Parameters		Goodness of Fit			
	*b*_1_	*b*_2_	SSE	*R* ^2^	Adjusted-*R*^2^	RMSE
DP2	3.932	0.06594	65.39	0.9779	0.9754	2.695
DP3-6	5.224	0.1547	22.26	0.9769	0.9744	1.573
Glu	0.951	0.04417	28.26	0.9307	0.923	1.772
Gal	0.01835	0.002243	28.26	0.9319	0.9244	0.5721

## Abbreviations

GRAS	generally recognized as safe
QPS	qualified presumption of safety
DP	degree of polymerization
DP2	disaccharides (lactose and non-lactose)
DP3-6	galacto-oligosaccharide fractions with a degree of polarization between 3 and 6
EMR	enzymatic membrane reactor
GOS	galacto-oligosaccharides
RMSE	root mean squared error
SSE	sum of squares due to error
STR	stirred tank reactor
UF ONPG	ultrafiltration *ortho*-Nitrophenyl-β-galactoside
**List of symbols**	
*c_b_*	bulk concentration of retained compounds (g·kg^−1^)
*c_E_*	enzyme concentration in reaction liquid (U·g^−1^)
*c_L_*	lactose concentration in feed (g·kg^−1^)
*c*_lim_	limiting concentration of retained compounds (g·kg^−1^)
*J*_lim_	permeate flux in Equation (5) (kg·h^−1^·m^−2^)
*k*	mass transfer coefficient (kg·h^−1^·m^−2^)
*P*	biocatalyst productivity (g·U^−1^·h^−1^)
*q*	permeate mass flow rate (kg·h^−1^)
*t*	operational time (h)
*Y*	yield of DP3-6 (*w*/*w*%)
**Greek letters**	
*τ*	residence time (h)
